# Efficient Uptake and Dissemination of Scrapie Prion Protein by Astrocytes and Fibroblasts from Adult Hamster Brain

**DOI:** 10.1371/journal.pone.0115351

**Published:** 2015-01-30

**Authors:** Jason R. Hollister, Kil Sun Lee, David W. Dorward, Gerald S. Baron

**Affiliations:** 1 Laboratory of Persistent Viral Diseases, Rocky Mountain Laboratories, NIAID, NIH, Hamilton, Montana 59840, United States of America; 2 Microscopy Unit, Research Technologies Branch, Rocky Mountain Laboratories, NIAID, NIH, Hamilton, Montana 59840, United States of America; Van Andel Institute, UNITED STATES

## Abstract

Prion infections target neurons and lead to neuronal loss. However, the role of non-neuronal cells in the initiation and spread of infection throughout the brain remains unclear despite the fact these cells can also propagate prion infectivity. To evaluate how different brain cells process scrapie prion protein (PrPres) during acute infection, we exposed neuron-enriched and non-neuronal cell cultures from adult hamster brain to fluorescently-labeled purified PrPres and followed the cultures by live cell confocal imaging over time. Non-neuronal cells present in both types of cultures, specifically astrocytes and fibroblasts, internalized PrPres more efficiently than neurons. PrPres was trafficked to late endosomal/lysosomal compartments and rapidly transported throughout the cell bodies and processes of all cell types, including contacts between astrocytes and neurons. These observations suggest that astrocytes and meningeal fibroblasts play an as yet unappreciated role in prion infections via efficient uptake and dissemination of PrPres.

## Introduction

Transmissible spongiform encephalopathies (TSEs), or prion diseases, are neurodegenerative diseases associated with the deposition of a partially protease-resistant form of prion protein termed PrPres. The infectious agent is thought to consist of either PrPres alone or in association with co-factor molecule(s) [1–8]. Although PrPres can be detected in some peripheral tissues [9,10], the main target for TSE disease is the central nervous system (CNS) where the most abundant PrPres deposits occur. It is of great interest to know how accumulation of PrPres damages the complex structure of the brain and which classes of cells play critical roles in the spread of infection and the development of neuropathology.

Different cell types of the brain have an intrinsic ability to propagate TSE infectivity. Immunocytochemistry studies 8 weeks after infection revealed that PrPres accumulation in astrocytes precedes astrocytosis and neuronal loss, suggesting a role for astrocytes at early time points post-infection [11]. Astrocyte-associated PrPres is present at clinical time points [12–15], but its origin is uncertain given that abundant neuron-derived PrPres is also present and may have been endocytosed by astrocytes. However, transgenic animals that express cellular prion protein (PrP^C^) only in neuronal cells or astroglial cells are susceptible to TSE infection, showing infectivity can replicate independently in these two cell types in vivo [16–18]. Cronier and colleagues demonstrated that isolated cerebellar granular neurons or cerebellar astrocytes exposed to low doses of infectivity steadily accumulate PrPres and amplify infectivity, indicating that neurons and astrocytes support TSE infection in cell culture [19]. Hamster glial cell cultures containing a mixture of astrocytes, oligodendrocytes, and microglia also propagate PrPres [20].

Despite the above observations, the question of whether CNS cells other than neurons play a role in the uptake and spread of PrPres during the course of prion disease has received little attention. This is surprising given the fact that astrocytes are abundant and possess numerous complex processes that make intimate contacts with neurons and other cells throughout the brain. Here, we used primary cells cultured from adult Syrian golden hamster brain to investigate early events occurring during acute exposure to exogenous PrPres. Our results show that CNS-derived astrocytes and fibroblasts internalize and disseminate PrPres with a much higher efficiency than neurons, suggesting these non-neuronal cell types may play a role in initiation of infection and spread of PrPres in the brain.

## Materials and Methods

### Antibodies and reagents

The following antibodies were used: mouse monoclonal anti-MAP2 (Millipore); chicken polyclonal anti-GFAP (Encor); rabbit polyclonal anti-Fibronectin (Abcam); mouse monoclonal anti-PrP antibodies 31C6 and mAb 132 [21]; mouse monoclonal anti-PrP antibody 6D11 (Covance); mouse-human chimeric recombinant Fab monoclonal anti-PrP antibody D13 (gift from Dennis Burton and Anthony Williamson) [22]; rabbit polyclonal anti-PrP antibody R20 gift from Byron Caughey [23]; DyLight 488-conjugated F(ab’)_2_ fragment of goat anti-human F(ab’)_2_ antibody (Jackson Immunoresearch); goat anti-mouse conjugated to Alexa Fluor 488 (Life Technologies); goat anti-rabbit conjugated to Alexa Fluor 488 (Life Technologies); and goat anti-chicken conjugated to Alexa Fluor 568 (Life Technologies). For labeling of subcellular compartments, fixable Dextran (10,000 MW) and Acetylated LDL conjugated to Alexa Fluor 488 (Dextran^A488^ and AcLDL^A488^), and LysoTracker Red (LT) were purchased from Life Technologies. Primary neuronal and glial cultures were isolated in HABG consisting of Hibernate A (Brain Bits) with 2% B27 (Life Technologies) and 0.5 mM GlutaMax (Life Technologies). Neuronal cultures were maintained in NABG consisting of Neurobasal A (Life Technologies) with 2% B27, 0.5 mM GlutaMax, 10 μg ml^-1^ gentamycin (Life Technologies), 5 μg ml^-1^ BDNF (Life Technologies), and 5 μg ml^-1^ bFGF (Life Technologies). Glial cultures were maintained in DMEM+ consisting of DMEM (Life Technologies) with 10% fetal calf serum (Life Technologies), 0.5 mM GlutaMax, and 10 μg ml^-1^ gentamycin.

### Ethics Statement

Animal experiments were conducted in an Association for Assessment and Accreditation of Laboratory Animal Care International (AAALAC)-accredited facility in accordance with animal welfare guidelines under animal study protocols (2010–30 and 2010–45) approved by the Animal Care and Use Committee of the Rocky Mountain Laboratories, National Institute of Allergy and Infectious Diseases, National Institutes of Health.

### Transmission electron microscopy

Samples were spotted onto freshly glow-discharged grids, stained with methylamine tungstate, and imaged as described in detail elsewhere [24].

### Primary cultures

Primary neuron-enriched cultures were prepared essentially as previously described [25,26] with modifications to culture conditions after plating as described in [27]. In our hands this improved neuronal survival and enrichment over the Brewer method. Briefly, an adult Syrian hamster was euthanized by carbon dioxide asphyxiation in accordance with guidelines set by the NIH Animal Research Advisory Committee, the NIH Office of Animal Care and Use, and AAALAC. The brain was submerged in HABG on ice and the meninges were removed. The cortical tissue from one hemispherical cortex was dissected and minced. The minced cortex was placed in 5 mL of cold HABG and then equilibrated at 30°C with gentle agitation. After equilibrating for 8 min, the liquid was replaced with 6 mL of 2 mg mL^-1^ papain (Worthington Biochemical) prepared in Hibernate A minus calcium (Brain Bits). The tissue was placed back at 30°C with gentle agitation for 30 min. After incubation, the liquid was replaced with 2 mL of HABG, and the cells were mechanically dissociated from the tissue by pipetting 10 times using a siliconized 9-inch Pasteur pipette with a fire-polished tip. After 1 minute to allow the remaining tissue pieces to settle to the bottom, the supernatant containing the cells was transferred to a fresh tube and the remaining tissue pieces were subjected to two more rounds of processing in 2 mL of fresh HABG. The total volume of the cell suspension was adjusted with HABG to 12 mL, and 6 mL of suspension was loaded per gradient onto two OptiPrep density gradients prepared with four steps (7.5%, 10.0%, 12.5% and 17.5%). The gradients were centrifuged at 800 x g for 15 min at room temperature. The neuron-enriched fraction was collected, which encompassed the 17.5% portion of the gradient. The cells were recovered by centrifugation, washed twice in HABG, and resuspended in 1–2 ml of NABG. The cells were counted, and 200,000 cells were seeded in poly-L-ornithine-coated glass bottom culture dishes (Willco dish; diameter 22 mm). After incubating for 1 h at 37°C, the dishes were rinsed with HABG to remove unattached cells and cell debris and then fed neuronal maintenance medium (NM) prepared as described below. After incubating for 2 days, half of the culture medium was replaced with fresh NM containing 6 μM cytosine arabinoside (AraC) to kill dividing cells. After incubating for 4–5 days, half of the medium was replaced with fresh NM without AraC. Cultures were typically utilized for experiments within 1–2 days of completing the AraC treatment but could be maintained for longer periods by replacing half of the culture volume with fresh NM every 3–4 days.

Non-neuronal cultures were prepared as described above for neuron-enriched cultures, except that the OptiPrep density gradient fractionation and AraC treatment steps were omitted. Briefly, one well of a BioCoat poly-D-Lysine 6-well plate (Becton Dickinson) was seeded with the cells obtained from 1 hemispherical cortex suspended in 3 mL of DMEM+. After incubating 1 h at 37°C, unattached cells and cell debris were removed by rinsing four times and then feeding with fresh DMEM+. The medium was replaced with fresh DMEM+ every 3 days until the culture reached confluence, usually after 7–10 days of incubation. The cells were then removed with trypsin, and an appropriate number of cells (150–1000) was seeded in poly-L-ornithine-coated glass bottom culture dishes (Willco dish; diameter 22 mm). Non-neuronal dishes were maintained by feeding fresh DMEM+ every 3 days.

Glial conditioned (GC) medium was prepared to support long-term maintenance of neuronal cultures essentially as previously described [27]. Briefly, one well of non-neuronal cells from a 6-well plate was seeded in DMEM+ in a 75 cm^2^ culture flask. Medium was replaced every other day until a confluent layer of cells had formed, at which time the growth medium was replaced with NABG containing 5 μM AraC. After 4 days, the AraC treatment was stopped by replacing the medium with NABG. AraC-treated cultures could be maintained for up to 1 month by replacing the growth medium every 3–4 days. To prepare GC medium, fresh NABG was incubated on AraC-treated cells for 24 h and then harvested, filtered and mixed with an equal volume of fresh NABG.

### Preparation of PrPres for infection of neuron-enriched and non-neuronal cultures

Hamsters were inoculated intracerebrally while under isoflurane anesthesia with 50 μl of 1% 263K-infected brain homogenate diluted in 2% fetal calf serum/phosphate-buffered balanced saline. Animals exhibiting clinical scrapie signs were euthanized by isoflurane overdose or carbon dioxide asphyxiation in accordance with guidelines set by the NIH Animal Research Advisory Committee and AAALAC. Brains were dissected from euthanized animals, rinsed in PBS, blotted on filter paper to remove excess blood, snap frozen in liquid nitrogen, and stored at -80°C until needed. DRM PrPres was prepared from the scrapie-infected brains as described previously [24]. The DRM PrPres was subsequently conjugated to Alexa Fluor 647-succinimidyl ester (Life Technologies) (263K^A647^) and characterized as described elsewhere [28].

### Infection of neuron-enriched and non-neuronal cultures

For infection of neuron-enriched or non-neuronal cultures, a fresh aliquot from a stock of 263K DRM PrPres conjugated to Alexa Fluor 647 (263K^A647^) was sonicated in a cup horn sonicator (Misonix model 3000) for 2 min on power 2 (average output power ~ 42W) with the experimenter manually holding the tube in the “sweet spot” to ensure optimal ultrasonic dispersion of the particles at these instrument settings. After sonicating, 25 ng was added directly to the culture supernatant, mixed by trituration, and the cultures were examined at 4–6 h, and later at 1, 3, and 5 days post-infection by live cell confocal microscopy.

### Immunofluorescence

Cells were fixed in a solution of 4% paraformaldehyde and 4% sucrose prepared in PBS (20 mM sodium phosphate and 130 mM sodium chloride, pH 7.4) for 15 min at room temperature followed by one rinse and two washes for 5 min each in 50 mM glycine (in PBS). The cells were then permeabilized with 0.1% Triton-X 100 (in PBS) for 10 min at room temperature followed by one rinse and three washes for 5 min each in PBS. To inhibit nonspecific antibody binding, cells were incubated for 1 h at room temperature in blocking solution (1% non-fat milk prepared in PBS). The following primary antibodies were diluted in blocking solution as indicated below and used to characterize the cells isolated from adult hamster brain: anti-MAP2 (1:1000), a marker protein for neurons; anti-GFAP (1:5000), a marker protein for astrocytes; and anti-fibronectin (1:100), a marker protein for fibroblasts. Cells were incubated overnight at 4°C or 1 h at room temperature with primary antibody and then rinsed once and washed three times for 5 min each in PBS. Cells were incubated for 1 h at room temperature with an appropriate secondary antibody conjugated to Alexa Fluor 488 or Alexa Fluor 568 diluted (1:1000) in blocking solution. Cells were rinsed once and washed three times for 5 min each in PBS before being observed by confocal microscopy.

Internalization of 263K^A647^ by cells in neuron-enriched and non-neuronal cultures was verified by PrPres-specific immunostaining with mAb 132 [29]. Cells pre-incubated with 263K^A647^ were fixed and permeabilized as described above. To reveal the epitope recognized by the antibody, cells were incubated in 5 M guanidine thiocyanate (GdnSCN; prepared in PBS) for 10 min at room temperature. After GdnSCN treatment, cells were washed in PBS, blocked for 1 h at room temperature, and then incubated with mAb 132 diluted in blocking solution overnight at 4°C with gentle rocking. Unbound primary antibody was washed away and bound primary antibody was labeled with anti-mouse IgG conjugated to Alexa Fluor 488 (Invitrogen, 1:1000). Cells were washed to remove unbound secondary antibody and then examined in PBS by confocal microscopy.

263K^A647^ preparations were also characterized by PrPres-specific immunostaining. The 263K^A647^ was diluted in PBS, sonicated under the conditions used for generating inocula for cell cultures, and then spotted onto glass coverslips and air-dried. The samples were then fixed and immunolabeled using PrPres-specific immunostaining conditions as described above with the exception that other anti-PrP antibodies were tested in addition to mAb 132 [epitope residues 119–127] including: 6D11 (epitope residues 93–109); D13 (epitope residues 96–104); and R20 (epitope residues 218–232). All antibodies showed specific, GdnSCN-dependent labeling of every 263K^A647^ particle in the sample. An isotype-matched anti-GFP mouse monoclonal antibody was also tested on GdnSCN-treated samples as a negative control.

### Labeling of subcellular compartments

Dextran^A488^ was used as a fluid-phase marker to label early and late endosomal/lysosomal vesicles. Cells pre-incubated with 25 ng of 263K^A647^ for 24 h were labeled with Dextran^A488^ (0.25 mg ml^-1^) for 16 h at 37°C. After incubating, the cells were washed to remove unincorporated Dextran^A488^, fed fresh DMEM, and incubated 1 h to chase endocytosed Dextran^A488^ to late endosomal/lysosomal vesicles. Cells were then fixed in paraformaldehyde (4% paraformaldehyde and 4% sucrose prepared in PBS) for 15 min at room temperature. To label early endocytic vesicles, cells pre-incubated with 263K^A647^ as described above were serum-starved (incubation in media without serum) for 20 min, and then Dextran^A488^ was added to the culture medium to a final concentration 0.5 mg mL^-1^. After 5 min of incubation, the unincorporated label was washed away, and the cells were fixed in paraformaldehyde (4% paraformaldehyde and 4% sucrose prepared in PBS) for 15 min. The labeled cells were examined by confocal microscopy.

AcLDL uptake in non-neuronal cells was visualized by incubating with AcLDL^A488^. The growth medium of cultures pre-incubated with 25 ng of 263K^A647^ for 3 days was supplemented with AcLDL^A488^ to a final concentration of 15 μg mL^-1^. After incubating for 4 h at 37°C, the cells were rinsed once and washed three times for 5 min each in DMEM+. Cells were then imaged by live cell confocal microscopy as described below.

To label acidic vesicles in neuron-enriched cultures, the cultures were treated with 25 ng of 263K^A647^ for 5 days before supplementing the culture medium with 1 μM LT. After incubating for 40 min at 37°C, the free dye was removed by rinsing the cells once and fed with GC medium. Cells were then imaged by live cell confocal microscopy.

### Image acquisition and analysis

Fixed and live cell confocal microscopy was performed with instruments as described previously [28,30] but with an Andor iXON3 897 camera and an ET700BP75 emission filter (Chroma) for images acquired on the Nikon LiveScan confocal. In some studies, a widefield DIC image was acquired with a CoolSnap HQ^2^ (Photometrics) camera, and the corresponding confocal image was overlaid onto the DIC image utilizing an image registration feature of Nikon Elements software. Sub-resolution Tetra-Speck fluorescent beads (Life Technologies) were used to facilitate image alignment. Select confocal images were deconvolved using Huygens Essential (SVI) or Nikon NIS Elements software but otherwise images were analyzed as previously described [30–32]. Time-lapse images were filtered using Gaussian, median, or advanced denoising filters (Imaris and Nikon NIS Elements software) as needed.

### Evaluation of the protease sensitivity of internalized 263K^A647^

To assess the protease sensitivity of intracellular 263K^A647^, non-neuronal cells were seeded in 6-well cell culture dishes and incubated with 100 ng of 263K^A647^. Treated cells were lysed at 4 h or 3 days post-exposure in PBS containing 0.5% Triton X-100 and 0.5% sodium deoxycholate. Seventy percent of each cell lysate was digested with Proteinase K (PK; 20 μg ml^-1^) for 30 min at 37°C. After termination of the PK digest with the addition of Pefabloc (2 mM), the PrPres was recovered by phosphotungstic acid precipitation [33]. Twenty percent of each cell lysate was precipitated with cold methanol without PK digestion to serve as an undigested control. Seventy nanograms of 263K^A647^ (the maximum 263K^A647^ possible in 70% of the treated cell lysate) was also digested and recovered by the same procedure to provide a control for input 263K^A647^. Protein pellets were solubilized in sample buffer (without running dye) by boiling and sonication before separation on 10% Bis-Tris NuPAGE gels in MES running buffer. Fluorescent protein samples must be examined using sample buffer without running dyes as these dyes are autofluorescent and can conceal the presence of low MW bands. After electrophoresis, the gels were incubated in Towbin for 5 min prior to visualization of 263K^A647^ by direct fluorescence on a Typhoon scanner (GE Healthcare).

## Results

### Analysis of fluorophore-labeled PrPres (263K^A647^)

**Fig 1:**
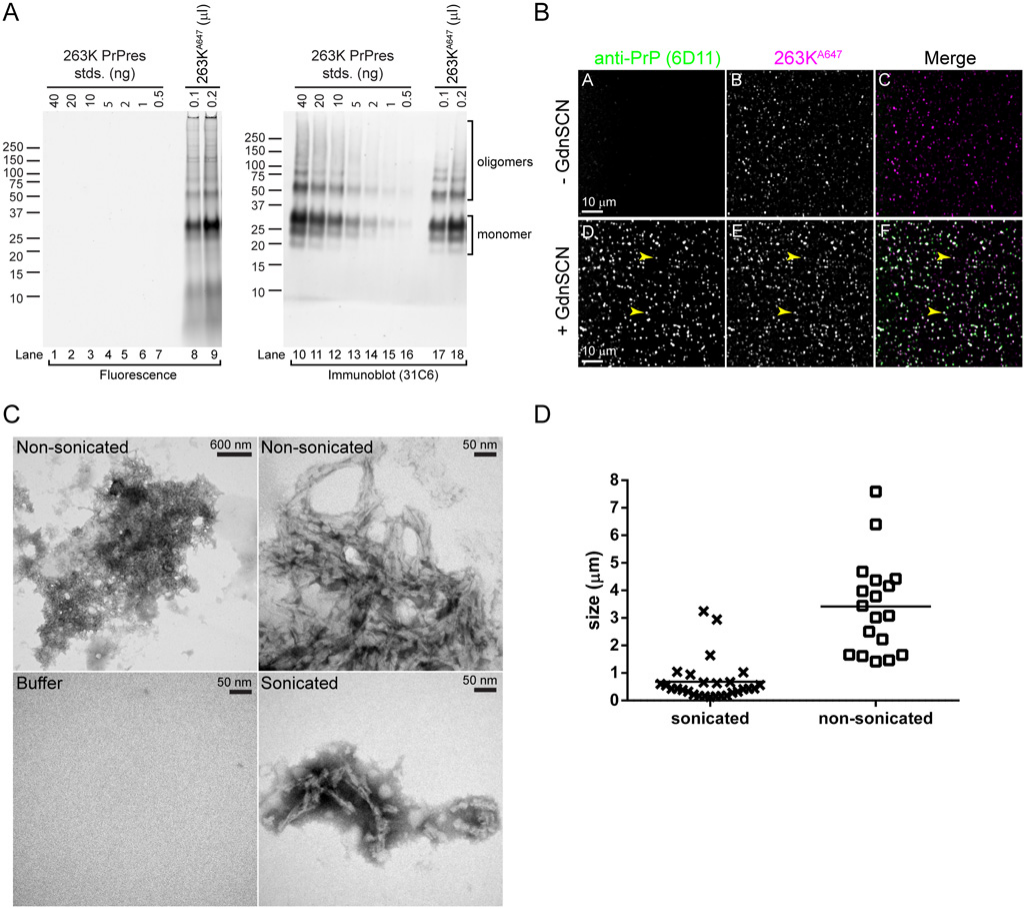
Characterization of 263K PrPres preparations covalently labeled with Alexa Fluor 647 (263K^A647^). (A) Fluorescent bands in 263K^A647^ are PrP. 263K^A647^ was resolved by SDS-PAGE. The gel was scanned to detect the fluorescent bands (Fluorescence) before immunoblotting with anti-PrP 31C6 (Immunoblot). Known quantities of unlabeled 263K PrPres were loaded as standards (lanes 1–7 and 10–16) for immunoblot quantitation of 263K^A647^. Fluorescent bands corresponding to 31C6-immunoreactive species constituted >85–90% of the total fluorescence. (B) Fluorescent particles in 263K^A647^ preparations all contain PrPres. Sonicated 263K^A647^ was spotted onto coverslips and immunostained with anti-PrP 6D11 with or without pretreatment with 3 M GdnSCN. All fluorescent 263K^A647^ particles (magenta) exhibited GdnSCN-dependent immunolabeling (green) indicative of PrPres. Merge, white indicates co-localization with equal signal from each channel. Arrowheads indicate examples 263K^A647^ particles with co-localized immunolabeling. (C) Electron micrographs of 263K^A647^ aggregates. Samples of 263K^A647^ before (Non-sonicated) and after (Sonicated) sonication were examined by TEM. Images of a non-sonicated aggregate acquired at low and high magnification are shown to allow visualization of the entire aggregate (upper left) and its composition (upper right). Buffer, buffer only control. (D) Analysis of 263K^A647^ aggregates in (C). The longest dimension of randomly selected aggregates in TEM images was measured and plotted. The mean size ± standard deviation for sonicated and non-sonicated 263K^A647^ aggregates was 0.683 μm ± 0.748 μm (n = 28) and 3.415 μm ± 1.676 μm (n = 18), respectively. These differences were statistically significant (P < 0.0001).

In order to visualize the uptake and trafficking of exogenous PrPres in primary adult-brain cell cultures, PrPres was purified from the brains of terminally ill 263K infected Syrian golden hamsters and covalently labeled with Alexa Fluor 647 (263K^A647^). Previous observations from our laboratory [28] and others [34,35] have shown that conjugation to various Alexa Fluor dyes still allows for PrPres internalization and does not significantly affect initiation of persistent infection in cell culture models of infection. Direct fluorescence analysis of 263K^A647^ resolved by SDS-PAGE (Fig. 1A, lanes 8–9) produced prominent fluorescent bands running between ~20 and 35 kDa with the uppermost band being most abundant. The ratio and pattern of bands observed reflected the typical glycoform pattern of the monomer form of PK-truncated 263K PrPres that has been reported in numerous studies [e.g. [24]]. Furthermore, the identity of the bands was confirmed by performing a western blot on the same gel with anti-PrP antibody 31C6, which showed a strong correlation between fluorescent and anti-PrP immunoreactive bands (Fig. 1A, lanes 8–9 vs. 17–18). Protein bands migrating at higher molecular weights were observed by both fluorescence gel scan and immunoblotting and represented SDS-resistant oligomer forms of PrPres. Quantitative analysis showed that fluorescence attributable to PrP based on fluorescent bands that also show 31C6 immunoreactivity accounted for >85–90% of the total fluorescence on the gel.

The fluorescent PrPres was also characterized by PrPres-specific immunolabeling of 263K^A647^ that was spotted onto glass coverslips. Since the epitopes of most anti-PrP antibodies are unavailable on PrPres aggregates without denaturation, GdnSCN-dependent antibody binding provides evidence of PrPres-specific immunoreactivity. We tested 4 different anti-PrP antibodies for GdnSCN-dependent immunostaining (mAb 132, 6D11, D13, and R20). These antibodies are directed against 3 distinct regions of PrP and use secondary antibodies directed against 3 different host species. One representative example of these immunolabeling experiments is shown in Fig. 1B. In every case, immunolabeling of fluorescent aggregates in 263K^A647^ was GdnSCN-dependent (Fig. 1B, panel A vs. D) and exactly co-localized with Alexa Fluor 647 fluorescence for every particle in the sample (Fig. 1B, panel D vs. E). No labeling was observed in GdnSCN-treated samples probed with an irrelevant isotype-matched anti-GFP monoclonal control antibody (S1 Fig.). Together, these data established the high purity of our 263K^A647^ preparation and verified they were suitable for the imaging experiments described below.

The size of PrPres particles can affect their uptake by cells [28], especially primary neurons [28,35]. We previously reported that sonication of Alexa Fluor-labeled PrPres improves uptake by primary neurons [28]. Therefore, 263K^A647^ preparations for inoculation into cultures were sonicated to generate small particles prior to addition to the cells. Sonicated 263K^A647^ preparations were examined by transmission electron microscopy to characterize the size of the particles and compared with non-sonicated controls to provide a point of comparison. Both types of samples contained clumps of short amyloid fibrils (Fig. 1C), but the average overall size of the clumps of fibrils was smaller for sonicated samples (Fig. 1D). This established that the sonication treatment was successful in generating a large population of smaller 263K^A647^ particles that based on our past experience were suitable for addition to primary neuronal cultures.

### Culturing neuronal and non-neuronal cells from adult hamster brain

**Fig 2:**
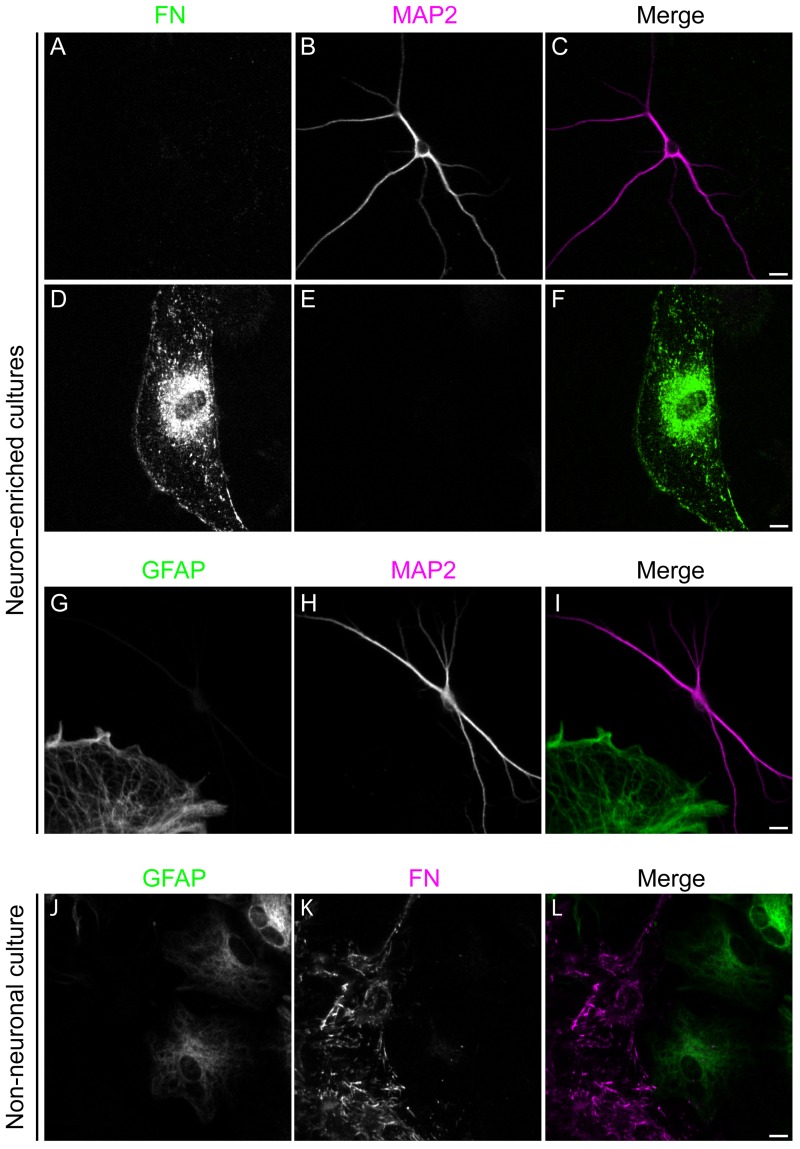
Identification of cell types isolated from adult Syrian hamster brain. Primary cells isolated from the brain of an adult Syrian hamster were cultured *in vitro* in NABG (Neuron-enriched) or DMEM+ (Non-neuronal), fixed and then stained for the presence of marker proteins for specific cell types. Neuron-enriched cultures were co-stained for the presence of the neuronal marker protein MAP2 (magenta) and the fibroblast marker fibronectin (FN, green) (shown in A-F) or MAP2 and the astrocyte marker protein GFAP (shown in G-I). Non-neuronal cultures were double stained for the presence of GFAP (green) and FN (magenta) (shown in J-L). Bar = 10 μm.

To investigate the uptake and trafficking of exogenous PrPres in adult brain cells we prepared two types of primary cultures from adult hamster brains, which we termed neuron-enriched and non-neuronal. Cultures with enriched neuronal cell populations were obtained by density gradient fractionation and selection for non-dividing cells by treatment with AraC. The neuron-enriched cultures were maintained in glial-conditioned Neurobasal A medium supplemented with B27, conditions that favor the long term survival of neuronal cells [26,27]. Thirty to sixty percent of the cells in the resulting cultures were stained with antibody against neuron-specific microtubule-associated protein, MAP2 (Fig. 2, panels A-C). The neurons regenerated multiple neuritic processes within four days and survived more than three weeks. MAP2-positive neuronal cells did not stain with anti-fibronectin or anti-GFAP (Fig. 2, panels A and G), which are markers for fibroblasts and astrocytes, respectively. Large non-neuronal cells represented 40 to 70% of the cells, and the majority of these cells stained with anti-GFAP or fibronectin antibody (Fig. 2, panels A and G). Although astrocytes adopted a variety of morphologies, all were readily distinguished from fibroblasts as astrocytes typically exhibited numerous projections while fibroblasts appeared as large, flat, crescent-shaped cells (Fig. 2 and see below). In >5 independent experiments, a combined total of only 2 Iba-1-positive cells were ever observed, indicating that microglia are extremely rare in the neuron-enriched cultures. A small percentage of cells failed to stain with antibodies against GFAP, fibronectin, Iba-1, and other glial cell markers but were easily distinguished from MAP2-positive neurons due to their glia-like morphology.

**Fig 3:**
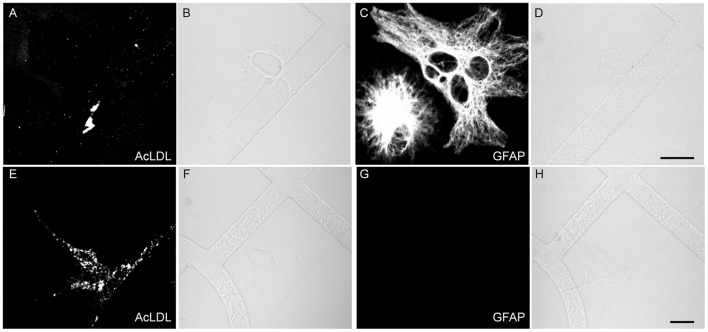
AcLDL uptake by meningeal fibroblasts but not by astrocytes. Non-neuronal cells seeded on gridded coverslip were incubated with AcLDL^A488^, imaged (A, C), and then fixed and stained with anti-GFAP (C, G). The location of the same cells imaged for AcLDL^A488^ was guided by the microscopic alphanumeric grid on the coverslip (B, D, F, H). Bar = 20 μm.

To prepare non-neuronal cultures, total cells derived from cortical tissue were grown in DMEM+ without performing the density gradient fractionation. After reaching confluence, the cells were trypsinized and reseeded at an appropriate cell density. Under these conditions, no MAP2-positive neuronal cells were found in the cultures. Instead, the cultures were composed predominantly of cells that appeared to be fibroblasts, based on the expression of high levels of fibronectin and astrocytes, distinguished by GFAP staining (Fig. 2, panels J-L). Antibodies against Iba-1 and PECAM-1 did not stain the non-neuronal cells indicating the absence of microglial and endothelial cells. Since astrocytes can express fibronectin, albeit at much lower levels than fibroblasts [36], we used an additional means of distinguishing astrocytes and fibroblasts. This involved live cell imaging of acetylated low density lipoprotein (AcLDL) uptake, the receptor for which is expressed on fibroblasts [37,38] but not astrocytes [39]. By sequential staining and imaging of individual cells that were first stained and imaged live with AcLDL^A488^ followed by fixation and immunostaining with anti-GFAP, we found that there was mutually exclusive labeling of cells with these two reagents in non-neuronal cultures (Fig. 3). This showed that AcLDL^A488^ uptake could serve as a fluorescent marker to distinguish fibroblasts from astrocytes by live cell imaging. The proportion of astrocytes and fibroblasts varied in each preparation, but the cultures usually contained more fibroblasts than astrocytes. Using this approach, three different classes of cells were cultured from adult hamster brain: neurons; astrocytes; and fibroblasts.

### Efficient uptake of 263K^A647^ by non-neuronal cells

**Fig 4:**
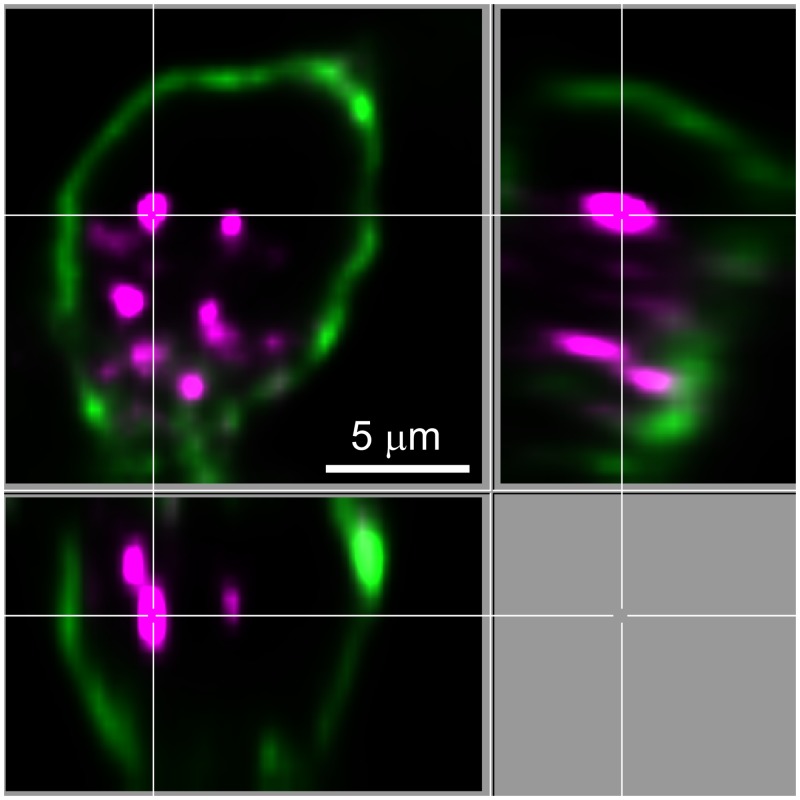
Neuronal cell body-associated 263K^A647^ particles are intracellular. To provide formal proof of the intracellular localization of 263K^A647^ particles (magenta) in cell bodies, neuronal cultures at 4 dpe were cooled to 4°C to inhibit endocytosis and treated with Alexa Fluor 488-tagged wheat germ agglutinin (green) to specifically label the cell surface. After labeling the cells were rinsed, fixed, and imaged by confocal microscopy. An orthogonal projection of a single optical slice from near the middle of the cell in a merged image is shown.

**Fig 5:**
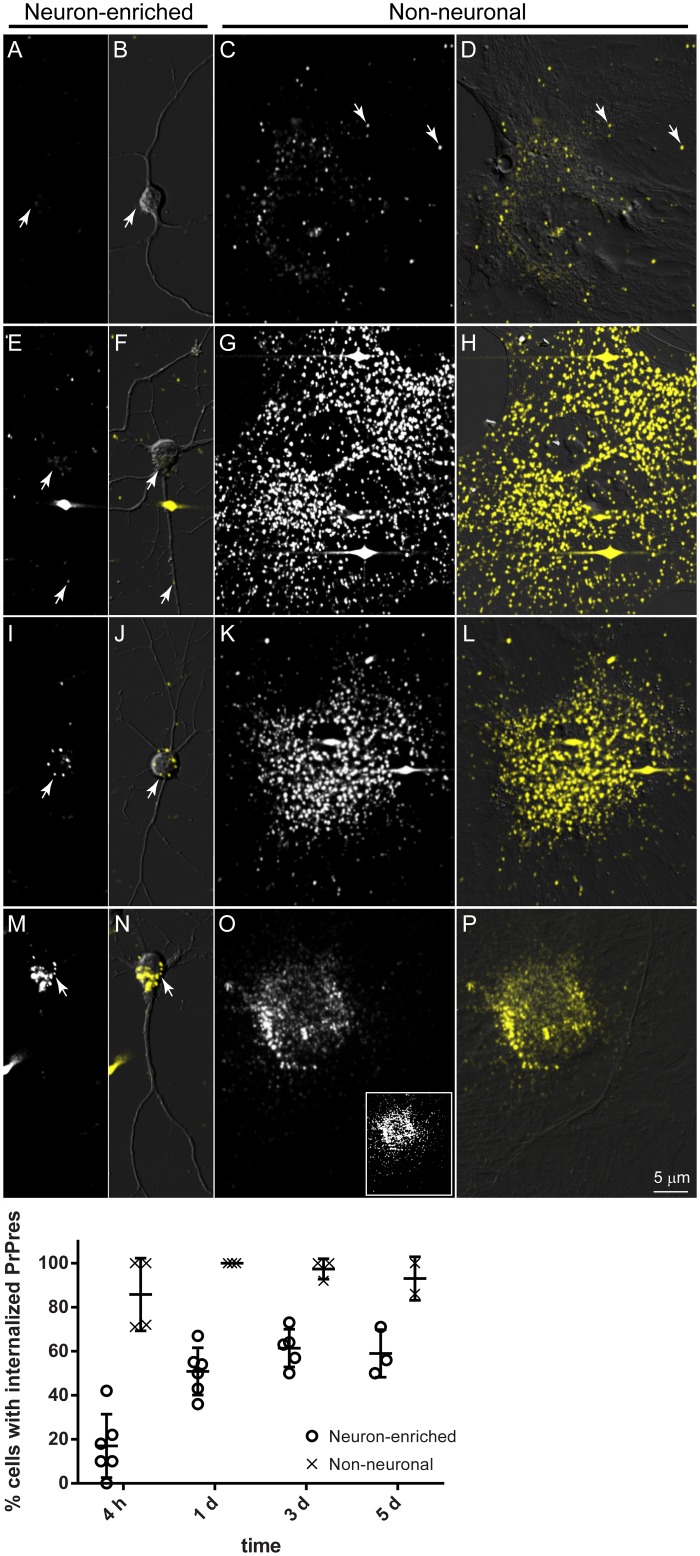
Non-neuronal cells internalize PrPres more efficiently than neurons. Neuron-enriched and non-neuronal primary cultures were incubated with 263K^A647^ and observed by live cell confocal microscopy at 4–6 h (A-D), 1 day (E-H), 3 days (I-L), or 5 days (M-P) post-exposure. After 4–6 h of incubation, fluorescent aggregates (arrows) were observed on cell surfaces in both cultures (A and C). The percentage of cells with internalized PrPres was significantly higher in non-neuronal cultures at all time points. Progressive accumulation of PrPres within non-neuronal cells necessitated imaging with about two-fold lower laser intensity at 5 dpe (panel O) to prevent detector saturation. Inset in panel O represents image of same field of view as panel O with same laser power setting used for panels C, G, and K. Panels B, D, F, H, J, L, N, and P show fluorescence images merged with the corresponding DIC images. Images A, E, I, and M are maximum intensity Z projections of optical sections totaling 3.5–6.5 μm and starting about 1.5 μm above the coverslip. Images C, G, K, and O are maximum intensity Z projections of optical sections totaling 1–4.5 μm and starting about 0.5 μm above the coverslip. The graph summarizes data accumulated over at least three independent experiments totaling between 39 and 67 cells observed at each time point for neuron-enriched cultures or at least two independent experiments totaling between 60 and 98 cells observed at each time point for non-neuronal cultures. All cells imaged were randomly chosen by initially viewing samples by DIC only (no fluorescence). Bars on graph represent mean ± standard deviation (SD).

**Fig 6:**
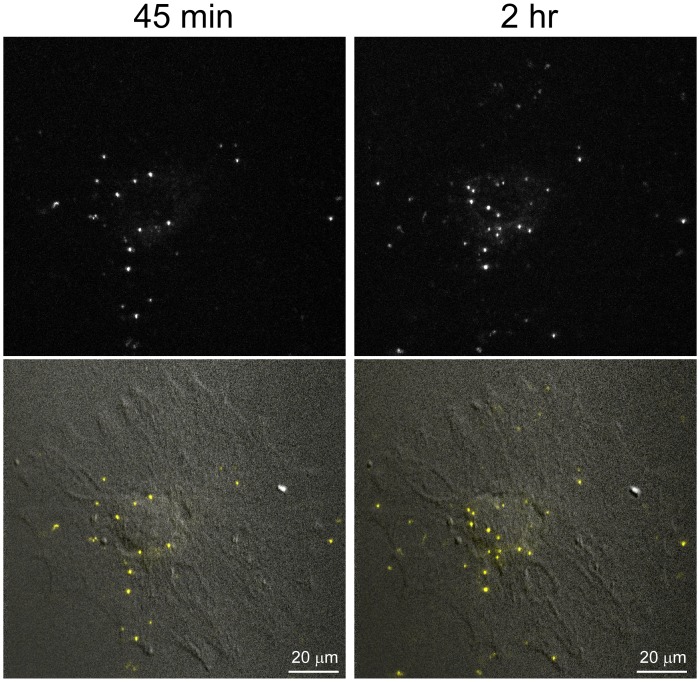
Analysis of 263K^A647^ uptake by non-neuronal cell at very early time points. Images acquired of the same non-neuronal cell at 45 min and 2 h after addition of 263K^A647^. A change in the distribution of fluorescent particles was observed over time, at least some of which were intracellular. This interpretation was supported by rapid time-lapse imaging of fluorescent PrPres trafficking in the cell at 2 hpe (S2 Video).

Fluorescent PrPres (263K^A647^) was added to neuron-enriched and non-neuronal cultures, and the cells were monitored by live cell confocal microscopy at selected time points for 5 days. Particles were classified as internalized on the basis of examining their location within individual optical slices for each cell and observing their movement within a cell in real time, such as in S1 Video, which shows an example of 263K^A647^ particles at 5 hours post-exposure (hpe) moving within the cell body and neurites of a neuron. In some instances, plasma membrane-specific staining with wheat germ agglutinin (WGA) was also used to identify intracellular particles (Fig. 4). At 4–6 hpe, 263K^A647^ was associated with cells in both types of cultures in the form of small fluorescent aggregates on cell surfaces (Fig. 5A and C). In addition to surface aggregates, we observed internalized fluorescent aggregates in small vesicle-like structures (Fig. 5C,G,I,K, M, and O), although the percentage of non-neuronal cells with internalized fluorescent aggregates was noticeably higher (86±16%) than neurons (17±14%) (Fig. 5, graph). We also verified normal endocytic function of the neurons using fluorescent cholera toxin (Ctx), which was readily internalized within 1 h of incubation (S2 Fig.). In non-neuronal cultures, numerous 263K^A647^ particles were found associated with the cells as early as 45 min post-exposure (Fig. 6). Over the next 75 min the distribution of the cell-associated fluorescent particles changed (Fig. 6). Analysis of individual Z slices and rapid time-lapse live cell imaging (S2 Video) of the movement of individual particles indicated that some particles were intracellular at 2 hpe.

The differences in 263K^A647^ uptake in neuron-enriched versus non-neuronal cultures persisted at later time points (Fig. 5, graph). All non-neuronal cells were very heavily labeled at 1 dpe with many fluorescent particles per cell that moved throughout the cells (S3 Video). The peak velocities with which the particles moved ranged from 0.18–2.0 μm/s (0.92 ± 0.35 μm/s, mean ± SD, n = 165). By 1 dpe the percentage of neurons (E) with internalized PrPres had increased about three-fold and remained about half the level observed in non-neuronal cells through 5 dpe (Fig. 5, graph). At later time points PrPres particles undergoing neuritic trafficking were observed with increased frequency (S4 Video). Progressive accumulation of PrPres within non-neuronal cells necessitated imaging with about two-fold lower laser intensity at 5 dpe to prevent detector saturation (Fig. 5, compare panel O to inset in O). PrPres aggregates per neuron increased over time similar to what was observed in non-neuronal cells but to a much lesser extent. Altogether, these observations demonstrated that non-neuronal cells were much more efficient than primary neurons at uptake and trafficking of PrPres with respect to both amount of PrPres/cell and percentage of PrPres-positive cells over time.

### Efficient 263K^A647^ uptake by astrocytes in neuron-enriched cultures

**Fig 7:**
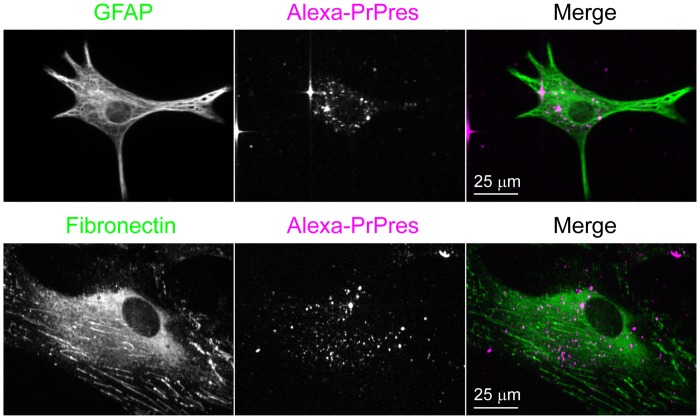
PrPres-positive non-neuronal cells present in neuron-enriched cultures are astrocytes and fibroblasts. Neuron-enriched culture was treated for 5 d with 263K^A647^ followed by immunostaining for astrocytes (GFAP) or fibroblasts (Fibronectin). Images correspond to single 0.5 μm optical Z slices.

**Fig 8:**
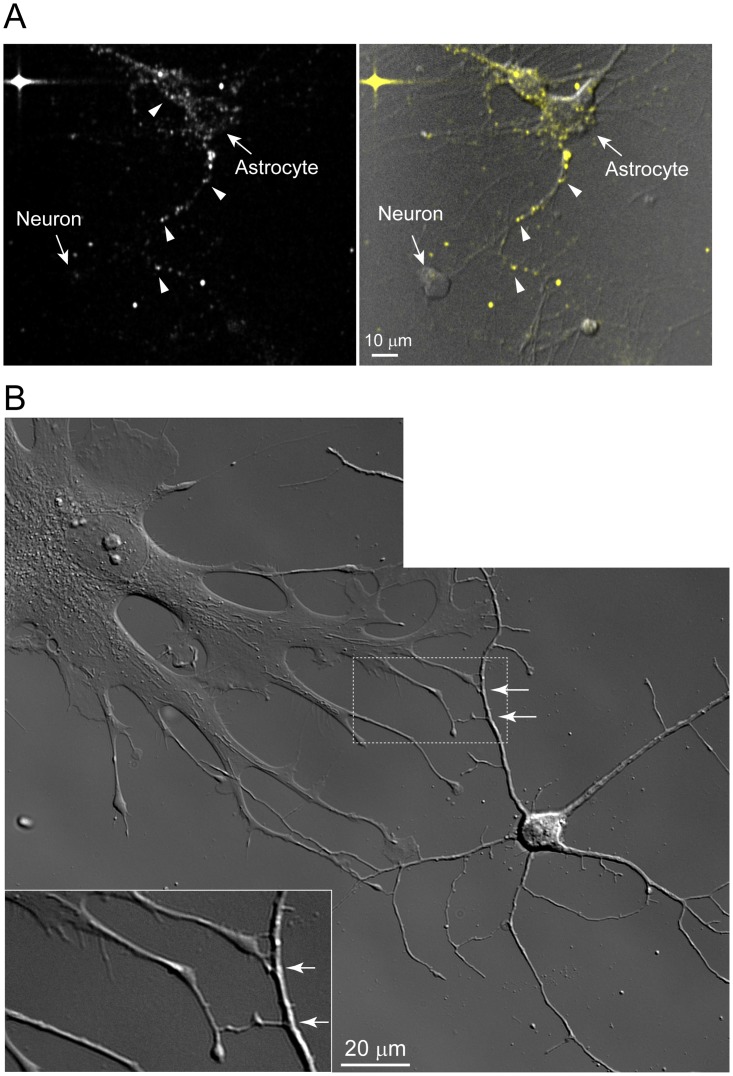
Astrocytes growing in neuron-enriched cultures efficiently internalize 263K^A647^ aggregates and produce long processes that often contact neurons. (A) Efficient internalization and dissemination of exogenous PrPres by primary astrocytes in neuron-enriched culture at 1 dpe. Astrocytes present within neuron-enriched cultures internalized PrPres at 1 dpe with similar efficiency to those grown in DMEM+. Vesicles carrying PrPres aggregates actively trafficked within the astrocyte processes. Neurons within the field of view appeared to make contact with PrPres-laden projections from astrocytes. Left panel, fluorescence channel. Right panel, merge of fluorescence and DIC channels. Arrowheads indicate 263K^A647^ particles in astrocyte cell bodies and projections. (B) Widefield DIC image of contact points between astrocytic projections and neurites. Some astrocytic projections appeared to be connected to neurites (arrows). Inset is a zoomed view of the dotted area.

Although the non-neuronal cells were cultured in different media than the neuronal cells additional control experiments indicated this did not account for the enhanced PrPres uptake by non-neuronal cells. For example, despite a slower growth rate, non-neuronal cells cultured in NABG still exhibited uptake of 263K^A647^ at a similar efficiency to those grown in DMEM+. More importantly, astrocytes (GFAP-positive) and fibroblasts (fibronectin-positive) that were present in neuron-enriched cultures also internalized 263K^A647^ (Fig. 7) and maintained the phenotype of enhanced 263K^A647^ uptake relative to neurons (Fig. 8A). In neuron-enriched cultures astrocytes more frequently adopted a morphology with longer processes (Fig. 8A) within which fluorescent PrPres particles were actively transported. Interestingly, astrocytic processes were often observed in contact with neurons, interactions that were more clearly visualized in DIC images where certain projections appeared to connect the two cell types (Fig. 8B, arrows). Thus, these data corroborated the observations from non-neuronal cultures and raised the possibility that contacts between neuron-astrocyte projections may be candidate routes for intercellular transfer of PrPres if it occurs.

### Internalized fluorescent particles are PrPres

**Fig 9:**
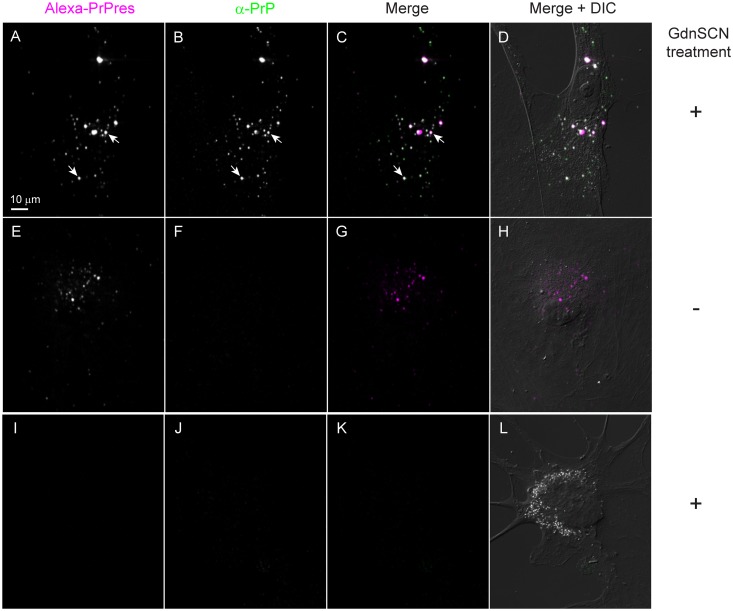
Fluorescent particles internalized by non-neuronal cells are PrPres. Non-neuronal cultures were treated with 263K^A647^ for 2 days and then subjected to PrPres-specific immunostaining with anti-PrP mAb 132. Internalized fluorescent aggregates (A and E) were specifically labeled with mAb 132 only when the samples were treated with GdnSCN (compare B to F). A negative control culture (I-L) treated with GdnSCN but not 263K^A647^ showed no fluorescence in the Alexa Fluor 647 channel (I) or immunostaining with mAb 132 (J). A merge of the Alexa-PrPres (magenta) and mAb 132 (green) channels is presented with (D, H and L) and without (C, G and K) an overlaid DIC image for each field of view. Merge, white indicates co-localization with equal signal from each channel.

Since certain cell types such as dendritic cells can degrade PrPres [40], we performed PrPres-specific immunofluorescence staining with mAb 132 anti-PrP antibody [29] on cells incubated with 263K^A647^ to confirm that the small fluorescent particles distributed throughout the cells in neuron-enriched (S3 Fig.) and non-neuronal (Fig. 9) cultures corresponded to intact PrPres. Virtually all of the Alexa Fluor 647-positive particles were detected by mAb 132 in a GdnSCN-dependent manner (Fig. 9A-H and S3A-H Fig.). In neurons this included particles present in both the cell bodies and neuritic projections (S3A and B Fig., arrows). No immunolabeling was detected in separate control cultures where 263K^A647^ was omitted, even with GdnSCN treatment prior to immunolabeling, demonstrating the PrPres-dependent specificity of immunolabeling (Fig. 9I-L and S3I-L Fig.). These results provided strong evidence that the small fluorescent particles distributed throughout the cells were intact PrPres.

### Characterization of 263K^A647^-positive vesicles

**Fig 10:**
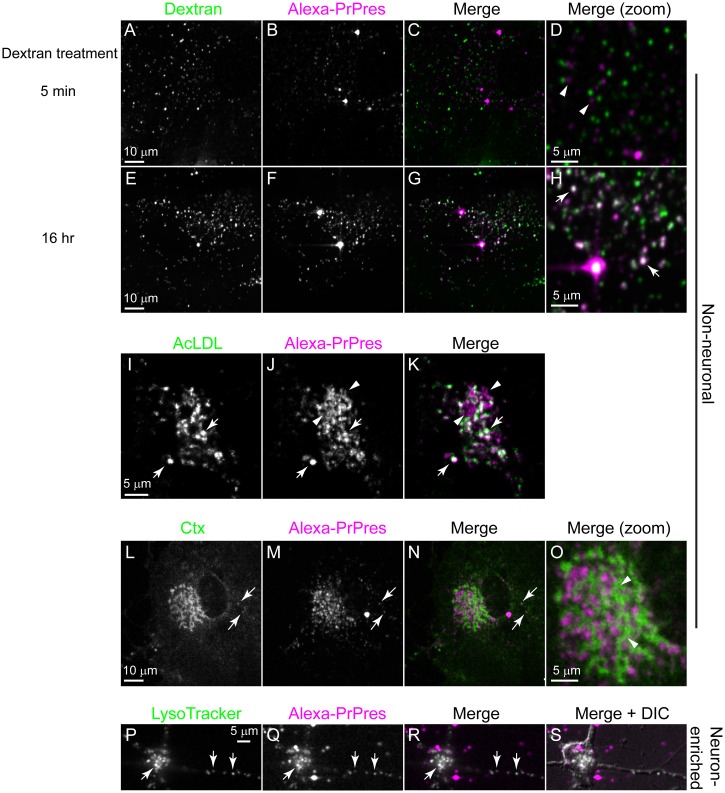
Internalized PrPres is trafficked to late endosomes/lysosomes. Neuron-enriched or non-neuronal cultures containing cells with internalized 263K^A647^ (B, F, J, M and Q) were incubated with the following markers: Dextran^A488^ for 5 minutes (A) as a marker for early endosomes or 16 h (E) as a marker for late endosomes/lysosomes; AcLDL (I) as a late endosomal/lysosomal marker in fibroblasts; Ctx (L) as a marker for rafts; and LysoTracker (P) as a marker for acidic organelles. Arrows indicate areas of co-localization. Arrowheads indicate areas without co-localization.

To identify the subcellular compartments where exogenous PrPres was trafficked in non-neuronal cells, non-neuronal cultures incubated with 263K^A647^ for 2–3 days were subsequently labeled with various organelle markers and imaged by confocal microscopy. First, we used pulse/chase labeling with the fluid phase endocytosis marker Dextran^A488^ to differentially label early endosomes or late endosomes/lysosomes. Early endocytic vesicles were labeled by incubation with Dextran^A488^ for 5 min. Late endosomes/lysosomes were labeled by overnight incubation with Dextran^A488^ followed by a 1 h chase in dextran-free medium. In both cases the samples were fixed at the end of the incubation period to prevent further trafficking of the internalized dextran during imaging. Dextran^A488^-positive late endosomal/lysosomal vesicles extensively co-localized with 263K^A647^-positive vesicles while dextran-positive early endosomes showed poor co-localization (Fig. 10A-D vs. 10E-H). Quantitation of co-localization of 263K^A647^ with dextran showed 82 ± 8.5% (mean ± SD, n = 6) co-localization after overnight dextran incubation and 24 ± 5.9% (mean ± SD, n = 6) after 5 min dextran incubation, differences which were statistically significant (P < 0.0001). Rapid time-lapse imaging of separate unfixed cultures labeled overnight with Dextran^A488^ showed that many of the vesicles positive for both dextran and 263K^A647^ were rapidly moving in real time throughout the cells (S5 Video). The peak velocities with which the particles moved ranged from 0.14–1.2 μm/s (0.74 ± 0.24 μm/s, mean ± SD, n = 88). Consistent with the dextran labeling results, there was strong co-localization of 263K^A647^ with LysoTracker, a marker that accumulates in acidified organelles (S4 Fig.). The above experiments showed that PrPres-containing vesicles dynamically interacted with fluid phase endocytic trafficking pathways.

Next, we examined the distribution of acetylated low density lipoprotein (AcLDL), which when pulsed for 4 h served as a fibroblast-specific late endosome/lysosome marker internalized by receptor-mediated endocytosis. Although many 263K^A647^-positive vesicles co-localized with AcLDL (Fig. 10I-K, arrows) there was a population that did not co-localize with AcLDL (Fig. 10I-K, arrowheads). Less co-localization was observed with the lipid raft marker cholera toxin (Ctx) (Fig. 10L-N, arrows), suggesting some internalized 263K^A647^ was not raft-associated. Quantitation of co-localization of 263K^A647^ with AcLDL showed 71 ± 12% (mean ± SD, n = 8) co-localization while there was 44 ± 11% (mean ± SD, n = 6) co-localization of 263K^A647^ with Ctx, differences which were statistically significant (P < 0.0009). All of the above were consistent with LysoTracker labeling of PrPres-treated neurons, where extensive co-localization (Fig. 10, P-S) and real-time rapid bi-directional transport of PrPres-positive vesicles was observed by live cell imaging (S6 Video). Collectively, the results demonstrated that 263K^AF647^ was primarily sorted to acidic late endosome/lysosome-like organelles in both neuronal and non-neuronal cells as has been reported for endogenously synthesized PrPres [23,41] and trafficking of fluorescent PrPres in SN56 mouse neuroblastoma cells [28].

### Internalized 263K^A647^ remains protease resistant

**Fig 11:**
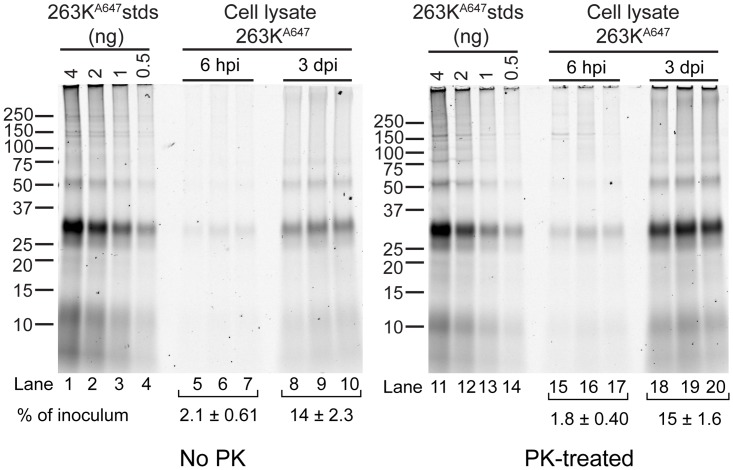
Biochemical characterization of internalized fluorescent aggregates in non-neuronal cells. Non-neuronal cells were treated with 263K^A647^ for the indicated times prior to lysis. No PK (lanes 5–10) samples correspond to 20% equivalents of cell lysates subjected to methanol precipitation. PK-treated (lanes 15–20) samples correspond to 70% equivalents of cell lysates subjected to PK digestion and PTA precipitation. Samples were analyzed by fluorescence SDS-PAGE. Lanes 1–4 and 11–14 contain dilution series of 263K^A647^ inocula that were processed identically to cell lysate samples but without application to the cells to serve as quantitation standards for input 263K^A647^. Numbers (% of inoculum) represent mean amount of PrPres recovered at each time point as a percentage of the corresponding inoculum ± SD (n = 3).

As mentioned above, certain cell types, such as dendritic cells, have been reported to degrade PrPres [40]. In addition, astrocytes also have the capacity to internalize and degrade amyloid aggregates [42–44]. Since internalized 263K^A647^ was localized to compartments positive for late endosomal/lysosomal markers, it was possible some of the internalized 263K^A647^ was trafficked to degradative compartments. To biochemically characterize the fluorescent material internalized by non-neuronal cells, non-neuronal cultures were incubated with 263K^A647^ and lysed after 6 h or 3 days as examples of early and late time points in the internalization process. Cell lysates were analyzed by SDS-PAGE and fluorescence gel scanning with and without proteinase K (PK) digestion. We observed no significant change in the banding profile of the 263K^A647^ in cell lysates (Fig. 11, lanes 5–10 and 15–20) compared with the inoculum (Fig. 11, lanes 1–4 and 11–14), suggesting that PrPres reached late endosomal/lysosomal compartments without significant modification. Consistent with observations by confocal microscopy, there was a substantial increase (~7 to 8-fold) in cell-associated 263K^A647^ from 6 h compared to 3 dpe (Fig. 11, lanes 5–7 vs. 8–10 and 15–17 vs. 18–20). Importantly, quantitative analysis of the gels showed similar results for PK-treated and untreated cell lysate-derived 263K^A647^ when normalized against corresponding PK-treated and untreated control inocula, indicating that the bulk of the internalized 263K^A647^ remained PK-resistant even at 3 dpe. Together with PrPres-specific immunostaining data (Fig. 1, 9 and S3 Fig.), this unequivocally demonstrated that the intracellular particles observed by confocal imaging corresponded to intact PrPres.

## Discussion

To assess the role of different cell types of the brain in acute uptake of the TSE agent, we isolated neuronal and non-neuronal cells from adult hamster brain and compared how these distinct cell types handle exogenous PrPres. Attempting to generate astroglial cultures, we grew adult brain-derived cells in the presence of FBS using an adaptation of classic techniques for culturing non-neuronal brain cells from neonatal animals, which employ FBS-containing media [e.g. [45]]. This likely contributed to the presence of GFAP-negative fibroblasts in these cultures. The presence of fibroblasts in adult astroglial cultures is a common observation [36] unless a specific procedure such as immunopanning is performed to eliminate those cells [46]. Although astrocytes and fibroblasts formed mixed cultures we found they could be distinguished in live cultures using a fluorescent conjugate of AcLDL (Fig. 3) and by immunolabeling after fixation (Fig. 2, panels J-L). Both of these cell types were present in neuron-enriched cultures, which allowed side by side comparison with neurons in the same culture. Astrocytes and fibroblasts were also the primary cell types in control non-neuronal cultures created by plating dissociated brain tissue directly into serum-free neuronal culture medium (NABG). These latter serum-free culture conditions may actually be more biologically relevant given recent evidence suggesting that gene expression profiles and morphological characteristics of astrocytes cultured in similar serum-free media more closely represent those of in vivo astrocytes when compared with classical serum-based astrocyte cultures [47,48]. Therefore, in this study we examined the uptake of PrPres by three different classes of primary cells: neurons; GFAP-positive astrocytes; and fibroblasts.

Although more technically challenging than embryonic or neonatal cell cultures, we focused on cell culture systems using cells from adult animals since most experimental TSE infections are initiated in mature animals and clinical disease and neuropathology occurs in adults due to the prolonged incubation periods. Also, there are differences in neurons and astrocytes from mature animals as compared to embryonic or neonatal animals. As neurons age several properties change compared to neurons derived from embryos including protein trafficking mechanisms, electrophysiological properties, glucose uptake, redox state, and sensitivity to the toxicity of Alzheimer’s Aβ peptide [49–53]. In astrocytes, differences have been observed in gene expression profiles [47,48,54], glutamate-dependent calcium signaling [55], and Aβ peptide uptake [43,44]. Thus, this is a novel cell culture system for TSE research that may better represent the physiological state of neurons as it exists during the course of prion infection in vivo.

Our results show that regardless of culture conditions brain-derived astrocytes and fibroblasts are more efficient than primary neurons at internalizing and trafficking PrPres. For instance, by 4–6 hpe an average of 86% of non-neuronal cells were positive for internalized PrPres compared to an average of 17% for neuronal cells with the latter reaching a maximum of about 60% by 3–5 dpe (Fig. 5). Since non-neuronal cells often occupied a larger surface area than neurons it is possible that this in part contributed to the higher PrPres uptake by non-neuronal cells. However, astrocytes cultured in NABG tended to have a much smaller surface area that in some cases was not much greater than neurons and even these astrocytes were strongly positive for PrPres at early time points (Fig. 8A). This argues that surface area alone cannot explain the difference in PrPres uptake efficiencies.

To determine the intracellular fate of exogenous PrPres, experiments labeling specific subcellular compartments were performed. Once internalized, the PrPres in all cell types was largely trafficked to compartments positive for markers of late endosomes/lysosomes (Fig. 10) where it persisted for several days (Figs. 5 and 11). Therefore, although the kinetics of uptake differed between cell types the PrPres appeared to be trafficked to similar subcellular compartments based on the markers used in this study. This was consistent with trafficking observed in SN56 mouse neuroblastoma cells [28] and other types of primary neuronal cells [35].

We observed that a smaller fraction of 263K^A647^ was destined for organelles labeled with the raft membrane marker cholera toxin (Fig. 10, Ctx, arrows). One interpretation of this data is that rafts have a small role in the PrPres internalization process. However, it is known that distinct raft markers can traffic through different intracellular organelles [56]. Moreover, different internalization rates between PrPres and cholera toxin could also result in poor co-localization, particularly if PrPres particles are only transiently localized to cholera toxin-positive compartments. In addition, in some fibroblasts we did observe a small population of PrPres that did not co-localize with AcLDL (Fig. 10, AcLDL, arrowhead). Further investigations are required to fully understand the potential role of rafts in uptake of exogenous PrPres.

Different from our findings, a related study reported internalization of fluorescent PrPres within 2 minutes in primary cultures of adult sensory and postnatal hippocampal neurons [35]. Uptake was clearly shown to be mediated by binding to low-density lipoprotein receptor-related protein 1 (LRP1), which also mediates the internalization of PrP^C^ [57,58]. The authors also reported that PrPres fibrils were transported to lysosomes that in neurites showed no movement during a 60 minute observation period [35]. By contrast, in primary adult hamster neurons we observed extensive rapid real-time trafficking of LysoTracker-labeled vesicles containing PrPres throughout cell bodies and neurites with net movement of individual particles in anterograde and retrograde directions (S6 Video). Among many differences between these studies such as cell types, concentration of PrPres applied to cells (we used ~200-fold lower concentration), and imaging methods, one key distinction is that Jen and co-workers used PrPres purified without PK treatment [35]. This was necessary for their purposes as PK treatment would result in the removal of N-terminal amino acids from PrPres that were shown to be required for PrP^C^ binding to LRP1 [58]. Hence, it is possible that N-terminal PrP residues may direct PrPres to different intracellular compartments that are not distinguished by labeling with LysoTracker. In our studies, we felt the use of PK-treated PrPres was important to ensure the preparation was of high purity and to avoid the removal of Alexa Fluor-labeled, N-terminal lysine residues of PrPres fibrils by cellular proteases as these residues are removed during processing of endogenously-synthesized PrPres [23,41]. Moreover, we used PrPres prepared using a new method developed in our lab that yields preparations with higher purity than can be obtained by commonly used methods [24]. Since purified PK-treated PrPres preparations remain infectious in cell culture and in vivo [28,34,59,60], this argues that PK-treated PrPres preparations are a valid reagent to investigate mechanisms of PrPres trafficking and TSE infection.

The contributions of various CNS cell types to the pathological processes occurring during TSE infection in vivo are not fully understood. There is evidence to suggest astrocytes may play a role in the disease process. Previous studies have demonstrated that astrocytes in primary culture [19,20,61] and in vivo [17,18] can support TSE infection. In clinical 263K-infected hamsters, PrPres was observed in a high percentage of hippocampal astrocytes [13]. Astrocytes positive for PrPres are present in clinical animals infected via peripheral routes with BSE, sheep scrapie, or HY transmissible mink encephalopathy, showing that astrocytes can acquire PrPres after prion entry into the brain via neurons [62–67]. In naturally-infected sheep disease-specific PrP can even accumulate to higher levels in astrocytes and glial cells than neurons [62–64]. PrPres accumulation in astrocytes has also been reported to occur as early as 8 weeks post-infection after intracerebral or cerebellar stereotactic inoculation [11]. Detection of PrPres in astrocytes preceded classical neuropathological changes associated with scrapie including PrPres accumulation in neurons and astrocytosis, suggesting astrocytes may be important at early stages of infection initiated via these routes [11]. This is consistent with the results of the present study.

After internalization by cultured primary non-neuronal cells, PrPres particles were transported throughout the cells and their processes and persisted through the course of our observation period as shown by multiple biochemical assays (Figs. 5, 9, and 11). This was particularly evident for astrocytes present in neuron-enriched cultures where cells more frequently adopted morphologies with some resemblance to astrocytes observed in vivo, such as cells with long, fine processes that made contact with other cells in the culture including neurons (Fig. 8). Remarkably, in vivo the highly complex processes of individual astrocytes can make contact with an average of four neuronal cell bodies (for cortical astrocytes) [68], hundreds of neuronal dendrites (for cortical astrocytes) [68], and an estimated 140,000 synapses (for hippocampal astrocytes) [69]. Single cortical astrocytes also envelop multiple dendrites of the same neuron [68] in addition to extending projections very near to but not overlapping with neighboring astrocytes [68,69]. The extensive intimate contacts between astrocytes alone and with neurons create opportunities for exchange of PrPres between these cells and in patterns that overlap with neuronal circuitry. When considered together with our observations, this raises the possibility that astrocytic transport may facilitate dissemination of PrPres within the brain, a subject that requires further investigation.

In addition to the typical scenarios already discussed above, non-neuronal cells could also contribute to TSE infection in the context of dura mater transplants. Dura mater of sporadic CJD and variant CJD patients has been shown to contain TSE infectivity and PrPres, respectively [70–72]. Transplantation of contaminated dura mater from donors with undiagnosed sporadic CJD is a major cause of iatrogenic CJD. Since fibroblasts are abundant in meninges and support TSE infection [73,74], meningeal fibroblasts are strong candidates for cells supporting prion propagation in this tissue. PrPres released from infected meningeal cells into the cerebrospinal fluid could seed prion propagation elsewhere in the brain. Moreover, astrocytes can make direct contacts with meningeal fibroblasts through their end-feet, especially in occasions of CNS injury [75]. During this interaction intercellular spread of PrPres could occur between the two cell types and transmit prions to the brain.

In summary, the results of the present study highlight a potential new role for astrocytes and fibroblasts as brain cell types participating in initiation of prion infection and spread of PrPres. Our data show that these cells, in addition to neurons, can transport PrPres particles over significant distances. Important questions to be addressed in future studies are whether intercellular spread of PrPres occurs in conjunction with this transport, what mechanisms are involved, and which population(s) of PrPres are involved (exogenous, newly-induced, or both).

## Supporting Information

S1 Fig Lack of immunostaining of 263K^A647^ with irrelevant antibody. Sonicated 263K^A647^ (magenta) was spotted onto coverslips, treated with 3 M GdnSCN, and immunostained (green) with mAb 132 or irrelevant isotype-matched negative control antibody (anti-GFP monoclonal). Fluorescent 263K^A647^ particles (magenta) were immunostained with mAb 132 but not anti-GFP. Merge, white indicates co-localization with equal signal from each channel. Bar = 5 μm.
(TIF)

S2 Fig Rapid uptake of cholera toxin by neurons. Neuron-enriched cultures were treated with Alexa Fluor 488-labeled cholera toxin B fragment for 1 hr at 37°C, washed, then imaged live by confocal microscopy. A) Maximum intensity Z projection of complete Z stack. B) Orthogonal projection showing internalized cholera toxin at optical plane near the middle of the cell. Bars = 5 μm.
(TIF)

S3 Fig Internalized fluorescent particles in neurons are PrPres. Neuron-enriched cultures were incubated with 263K^A647^for 5 days then subjected to PrPres-specific immunostaining with anti-PrP mAb 132. Internalized fluorescent aggregates (A and E, arrows) in neurons showed GdnSCN-dependent immunolabeling with mAb132 that is indicative of PrPres (compare B to F). A negative control culture (I-L) treated with GdnSCN but not 263K^A647^ showed no fluorescence in the Alexa Fluor 647 channel (I) or immunostaining with mAb 132 (J). A merge of the Alexa-PrPres (magenta) and mAb 132 (green) channels is presented with (D, H and L) and without (C, G and K) an overlaid DIC for each field of view.
(TIF)

S4 Fig Co-localization of 263K^A647^ and LysoTracker in non-neuronal cells. Non-neuronal cells were incubated with 263K^A647^ for 2 days, stained with LysoTracker Green, washed, and then imaged live by confocal microscopy. A single optical section is shown. Arrows indicate areas of co-localization. Bar = 10 μm.
(TIF)

S1 Video Example of 263K^A647^ trafficking within neuron at 5 hpe. Arrows delineate three separate neurites in which 263K^A647^ particles were trafficking towards and away from the cell body. Particles are observed leaving (top neurite) and entering (neurite at 10 o’clock) the cell body as well as moving within the cell body. A focal plane near the coverslip is shown.
(MP4)

S2 Video 263K^A647^ particle trafficking in astrocyte in non-neuronal culture at 2 hpe. Rapid timelapse live cell imaging of the cell shown in Fig. 6 (2 hr time point) showed 263K^A647^ particles moving within the cell as early as 2 hpe. Arrows highlight two selected particles as examples.
(MP4)

S3 Video Intracellular trafficking of PrPres in non-neuronal cell with abundant internalized PrPres at 1 dpe. Timelapse of single optical section about 1.2–1.6 μm above the coverslip.
(MP4)

S4 Video PrPres trafficking in neurite and cell body of primary neuron at 3 dpe. Video is a rapid timelapse showing 263K^A647^ transport within neuritic projection. The fluorescent channel was superimposed on a DIC image of the neuron to illustrate the position of the cell and the neurite. Shows PrPres particle exhibiting net movement within neurite towards the cell body (upper 3 arrowheads) and particle moving from cell body into neurite (bottom arrowhead near cell body).
(MP4)

S5 Video 263K^A647^ co-trafficking with Dextran^A488^ in non-neuronal cell. Cells were imaged after sequential treatments with 263K^A647^ (2–3 days) and Dextran^A488^ (16 hr) as described for Fig. 10 (E-H). Scale bar, 10 μm.
(MP4)

S6 Video 263K^A647^ co-trafficking with LT in neuron shown in Fig. 10 (P-S). 263K^A647^-containing vesicles were virtually all positive for LT. These vesicles exhibited net movement towards and away from the cell body within neuritic projections (white arrowheads) and moved into and out of the cell body (yellow arrowheads).
(MP4)
