# Supplementary figures and images for: Efficient Uptake and Dissemination of Scrapie Prion Protein by Astrocytes and Fibroblasts from Adult Hamster Brain
Source: PLoS One. 2015 Jan 30;10(1):e0115351. doi: 10.1371/journal.pone.0115351 (PMC4311963; doi:10.1371/journal.pone.0115351)

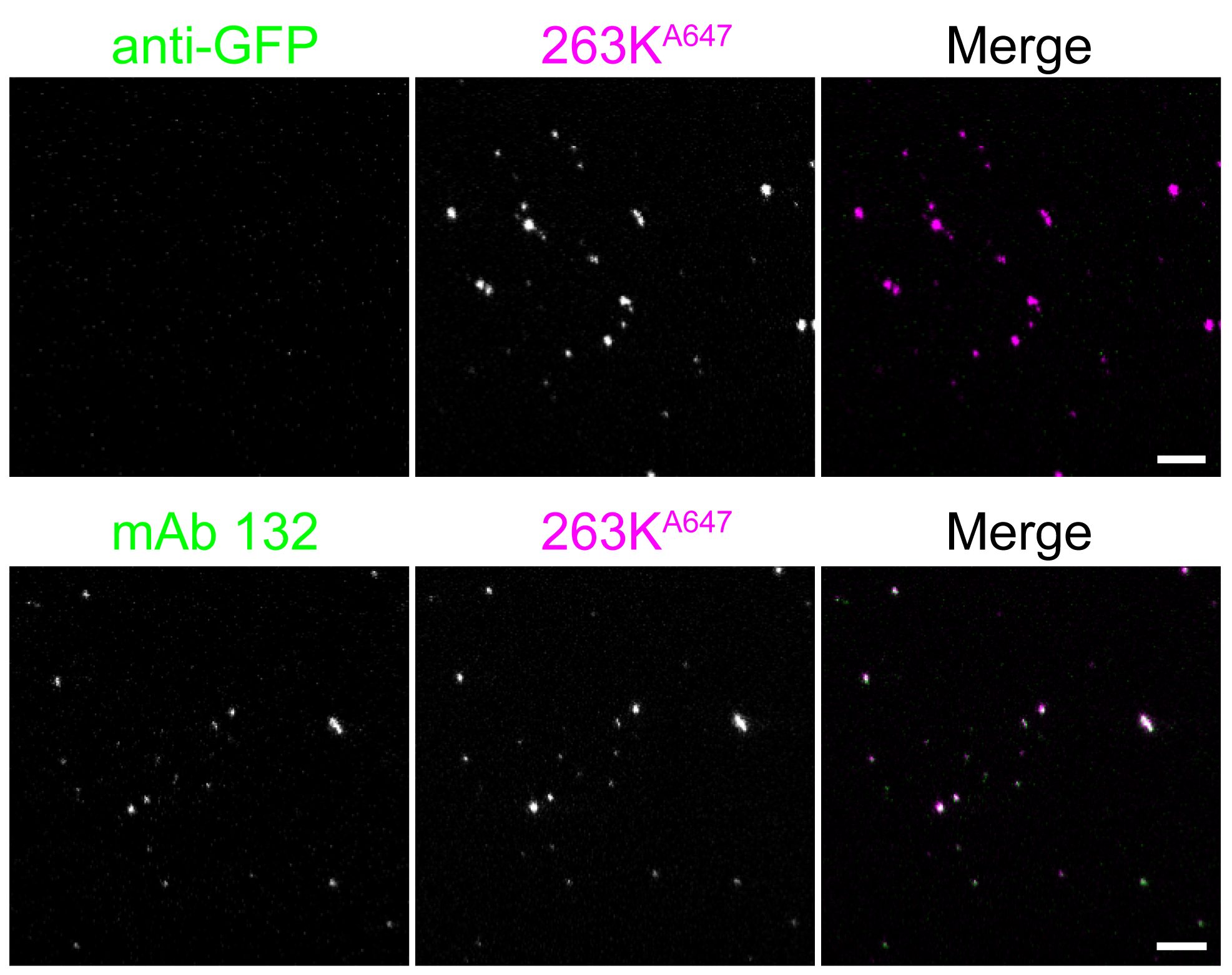

Supplement: S1 Fig — Sonicated 263KA647 (magenta) was spotted onto coverslips, treated with 3 M GdnSCN, and immunostained (green) with mAb 132 or irrelevant isotype-matched negative control antibody (anti-GFP monoclonal). Fluorescent 263KA647 particles (magenta) were immunostained with mAb 132 but not anti-GFP. Merge, white indicates co-localization with equal signal from each channel. Bar = 5 μm. (TIF) [file pone.0115351.s001.tif]

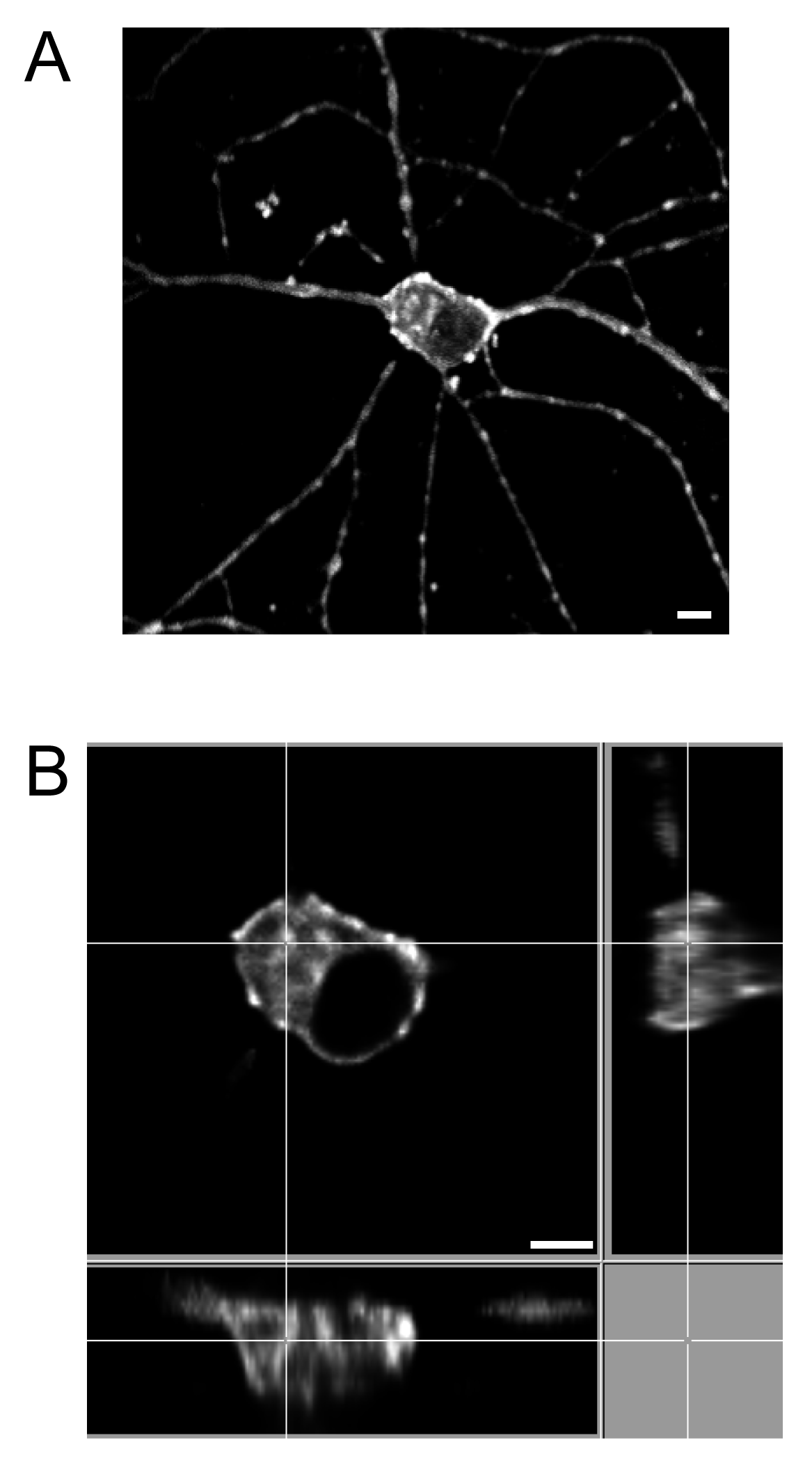

Supplement: S2 Fig — Neuron-enriched cultures were treated with Alexa Fluor 488-labeled cholera toxin B fragment for 1 hr at 37°C, washed, then imaged live by confocal microscopy. A) Maximum intensity Z projection of complete Z stack. B) Orthogonal projection showing internalized cholera toxin at optical plane near the middle of the cell. Bars = 5 μm. (TIF) [file pone.0115351.s002.tif]

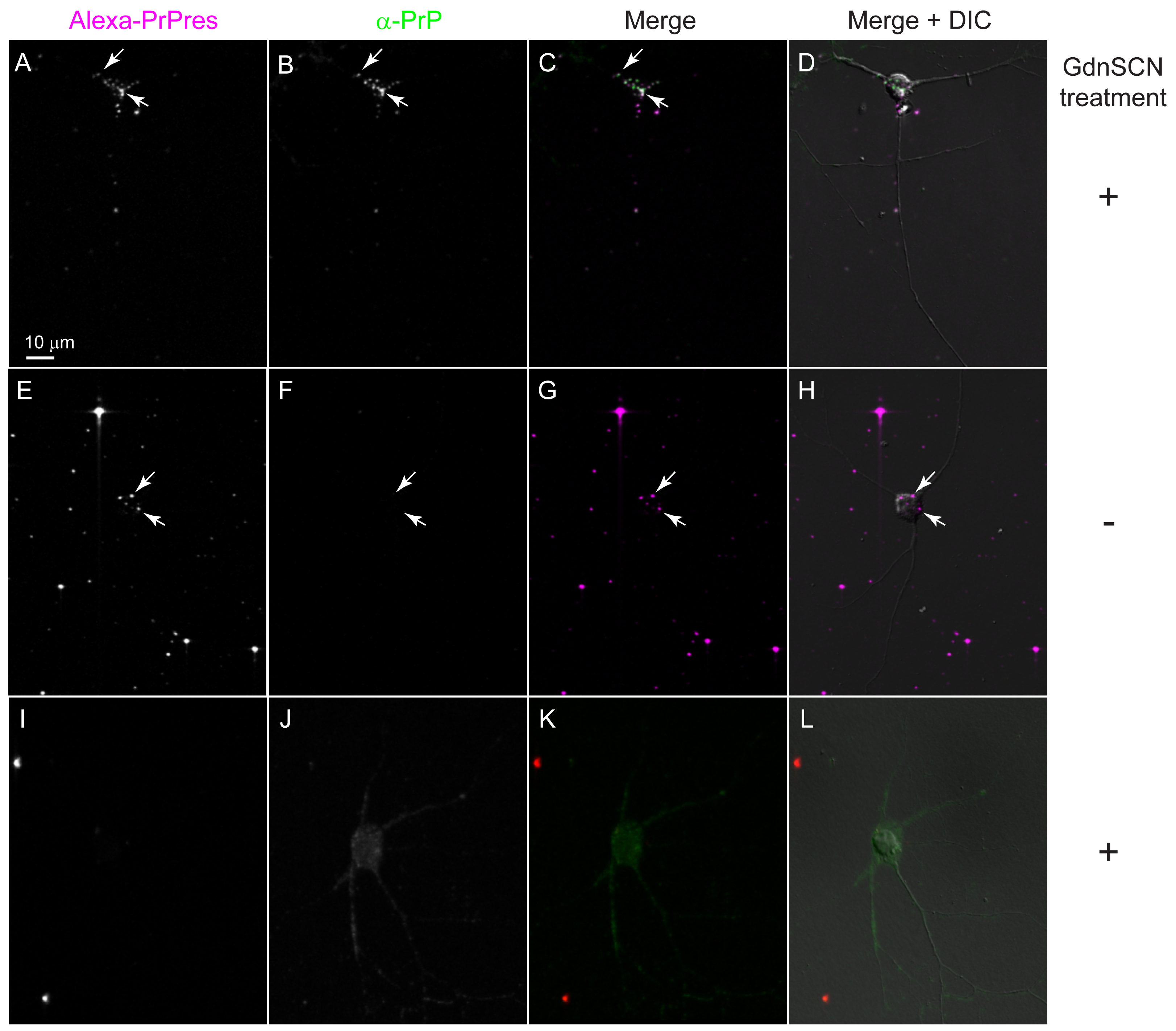

Supplement: S3 Fig — Neuron-enriched cultures were incubated with 263KA647for 5 days then subjected to PrPres-specific immunostaining with anti-PrP mAb 132. Internalized fluorescent aggregates (A and E, arrows) in neurons showed GdnSCN-dependent immunolabeling with mAb132 that is indicative of PrPres (compare B to F). A negative control culture (I-L) treated with GdnSCN but not 263KA647 showed no fluorescence in the Alexa Fluor 647 channel (I) or immunostaining with mAb 132 (J). A merge of the Alexa-PrPres (magenta) and mAb 132 (green) channels is presented with (D, H and L) and without (C, G and K) an overlaid DIC for each field of view. (TIF) [file pone.0115351.s003.tif]

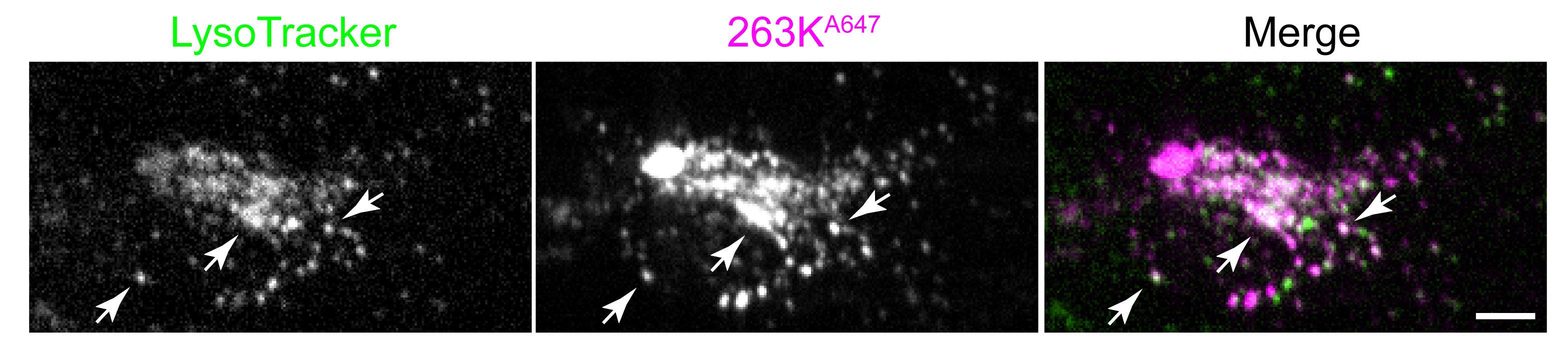

Supplement: S4 Fig — Non-neuronal cells were incubated with 263KA647 for 2 days, stained with LysoTracker Green, washed, and then imaged live by confocal microscopy. A single optical section is shown. Arrows indicate areas of co-localization. Bar = 10 μm. (TIF) [file pone.0115351.s004.tif]
